# Economic burden of varicella in Bangkok, Thailand: A multicenter medical chart review study

**DOI:** 10.1371/journal.pgph.0003099

**Published:** 2024-06-12

**Authors:** Salome Samant, Manjiri Pawaskar, Sujittra Chaisavaneeyakorn, Supitcha Kamolratanakul, Sujane Limpadanai, Bianca Jackson, Jennifer Stephens, Isaya Sukarom, Kulkanya Chokephaibulkit

**Affiliations:** 1 Merck & Co., Inc., Rahway, New Jersey, United States of America; 2 Ramathibodi Hospital, Mahidol University, Bangkok, Thailand; 3 Tropical Medicine Hospital, Mahidol University, Bangkok, Thailand; 4 Mission Hospital, Bangkok, Thailand; 5 OPEN Health, Bethesda, Maryland, United States of America; 6 MSD Thailand, Bangkok, Thailand; 7 Siriraj Hospital, Mahidol University, Bangkok, Thailand; University of Cape Town, SOUTH AFRICA

## Abstract

Our multicenter, medical chart review, cost-of-illness study used a micro-costing approach to evaluate the economic burden associated with varicella in Bangkok, Thailand, from a societal perspective. We reviewed medical charts of adults and children with a primary diagnosis of varicella (2014–2018) from 4 hospitals in Bangkok. Reported healthcare resource utilization and missed school or workdays were extracted from medical charts. Mean direct, indirect, and total costs per patient were estimated for overall, adult, and pediatric patients (2020 USD). Of the 200 children and 60 adults, 99.6%, 5.4%, and 5.4% had a varicella-related outpatient visit, emergency department visit, and hospitalization, respectively. The mean direct medical cost was 33 USD for pediatric and adult patients. The mean cost of outpatient visits (8 vs 13 USD, *P*<0.001) and medications (7 vs 9 USD, *P*<0.001) was significantly lower among pediatric patients. Forty-eight children reported a mean of 5.8 school days lost, and 32 adult patients reported a mean of 7.4 workdays lost. The mean total cost per varicella patient was 89 USD, with the mean total cost higher for adult than pediatric patients (145 vs 72 USD, *P*<0.001). Indirect cost accounted for 63% of the total cost per patient (54% for pediatric patients and 77% for adult patients). There is a substantial economic burden associated with patients seeking varicella-related healthcare in Thailand, including considerable indirect costs.

## Introduction

Primary infection with the varicella-zoster virus causes varicella or chickenpox, which usually presents as fever, malaise, headache, and an itchy, vesicular skin rash. In the absence of vaccination, almost everyone acquires the disease over their lifetime, usually during childhood. Infants, adults, and immunocompromised persons are at higher risk of developing complications from varicella, including pneumonia and skin infections [1,2].

The annual incidence of varicella in the general population in Asia Pacific in the absence of universal varicella vaccination (UVV) programs varies depending on the quality of surveillance and other factors, with the highest estimated pre-UVV annual incidence (2,530 per 100,000 persons) reported in Taiwan, which has a robust national surveillance system [3]. The implementation of UVV has led to a substantial decrease in varicella incidence, healthcare resource utilization (HCRU), and costs associated with varicella in various countries [1,4–8].

Previously, a multi-country study (MARVEL) was conducted to assess HCRU and costs associated with varicella in 5 middle-income countries without UVV (Hungary, Poland, Argentina, Mexico, and Peru, 2009–2016) [9]. That medical chart review study found that varicella was associated with substantial HCRU and financial burden on health systems. Systematic literature reviews have found limited data on the economic burden of varicella in the general population in the Asia-Pacific region, with data coming from older studies conducted in high-income countries like Australia, New Zealand, Taiwan, Hong Kong, and Singapore [3,10–19].

There are few studies on the economic burden of varicella in Thailand. One study examined resource utilization and direct medical costs incurred by 101 children hospitalized for varicella in a Thai hospital from 2007 to 2011. That 2014 study reported median hospital charges of $330 USD, with a median hospital stay of 6 days [20]. Another study assessing a varicella outbreak in a Thai hospital where 140 healthcare workers and 18 patients were exposed found the cost of managing the outbreak to be $23,087 [21].

In Thailand, a tropical country without UVV, considerable varicella burden is seen among adolescents and adults, unlike in temperate countries (where >90% of varicella cases occur before 10 years of age), probably due to the impact of climate, population density, and risk of exposure [1,22–24]. This is supported by a Thai seroprevalence study, where only 27% of children aged 12 years or younger had seroprotection against varicella versus all adults by age 50 years [23]. Varicella is usually more severe in adults, with those aged ≥45 years having 4–50 times greater risk of hospitalization [1,4]. Hence, it is important to assess the economic burden of disease in Thailand in both pediatric and adult patients to capture the full financial burden of varicella in the country.

The purpose of this study was to estimate the economic burden of varicella in pediatric and adult patients seeking healthcare in Bangkok, Thailand. We also compared the costs of varicella in pediatric versus adult patients.

## Methods

This was a multicenter cost-of-illness study conducted from a societal perspective using a micro-costing approach to evaluate the economic burden associated with varicella. We reviewed the medical charts of 200 children (aged <18 years) and 60 adults (aged ≥18 years) with a primary diagnosis of varicella, randomly selected from 4 sites in Bangkok, Thailand, over 5 years (January 1, 2014-December 31, 2018): Siriraj Hospital (n = 100), Ramathibodi Hospital (n = 80), Hospital for Tropical Diseases (n = 50), and Mission Hospital (n = 30). The 4 hospitals, all public except Mission Hospital, serve the Bangkok region, which is home to 22% of the Thai population [25]. Patients were randomly selected based on month and year of index date to account for any seasonal variation in costs. Patients admitted to the hospital for varicella were classified as inpatients, with the others classified as outpatients. Exclusion criteria for this study included a diagnosis of herpes zoster or prior varicella vaccination. Additional details on study design, clinical outcomes, and medication use are published elsewhere [26].

Data on patient characteristics and HCRU were abstracted from each patient’s medical record by study physicians starting August 28, 2020, using case report forms and physician notes from the time of varicella diagnosis until disease resolution or the last contact date. Additionally, physicians were asked to complete surveys to collect information on practice characteristics, overall healthcare-seeking behavior of varicella patients, and unit costs associated with different services (e.g., inpatient hospitalizations [per day], outpatient office/clinic visits, emergency department visits, tests and procedures, and medications) provided to varicella patients at their hospital. We also used secondary data sources (published literature and Thai government websites) to obtain unit costs, as needed (S1 Table). Unit costs for prescriptions and over-the-counter medications were obtained from the Drug and Medical Supply Information Center maintained by the Thai Ministry of Public Health, which contains a reference price of drugs and medical supplies used by government hospitals for procurement [27]. We did not include staff costs or consultation time in our analysis. Missed school days and workdays were abstracted from physician notes wherever available. Costs were reported in both 2020 Thai bahts (THB) and US dollars (USD) (1 THB = 0.032 USD); conversion to 2020 costs was done using the Consumer Price Index for Thailand [28]. Key outcomes included HCRU and mean direct, indirect, and total (direct + indirect) costs for pediatric and adult patients. We also tried to estimate the potential annual national burden of varicella infection in Thailand.

HCRU was assessed using the number and proportion of patients utilizing each resource and the duration of use. Direct medical costs were determined at the patient level by multiplying the number of resources used per patient by the unit cost of each resource, from either published sources [27] or from physician surveys at the study sites, using a bottom-up (micro-costing) cost approach. Direct medical costs included the cost of hospitalization, emergency department and outpatient visits, consultations with specialists, tests, procedures, and prescribed medications. Conservative assumptions were made regarding the frequency and dosage of medications if such information was missing.

Productivity loss was estimated using a human capital approach [29]. The indirect cost analysis included only the reported productivity losses abstracted from patient records. For children, we assumed that the reported school days lost would equal the workdays missed by that child’s parent (i.e., caregiver productivity loss). For hospitalized patients, the time missed was assumed to be equal to the length of the hospitalization in the absence of a note in the medical records. Reported productivity losses for adults (missed workdays) and children (missed school days, assumed to result in caregiver productivity loss) were multiplied by the average daily wage (28 USD/884 THB) for the Bangkok region [28,30] to calculate indirect costs for each patient. A sensitivity analysis was done using a minimum daily wage of 11 USD/331 THB for the Bangkok region [31]. Additionally, in a second sensitivity analysis for indirect costs, we assumed zero productivity loss among caregivers of pediatric patients because of alternate caregiving arrangements in place such as a non-working parent/grandparent at home.

The total cost of varicella was estimated for each patient by adding the direct and indirect costs incurred. As an exploratory analysis, we also estimated the national burden of varicella in Thailand based on these data as well as published literature (S1 Fig).

Descriptive statistics were produced for all study variables using SAS software version 9.4 (SAS Institute Inc., Cary, NC, USA). Outcomes were compared between pediatric and adult populations using the chi-square test, Fisher’s exact test, or Wilcoxon rank sum test as appropriate, with a cutoff of *P*<0.05 considered statistically significant.

### Ethics statement

This study was approved by CREC: Central Research Ethics Committee (National Research Council of Thailand; #2563; June 15, 2020) and the local ethics committee at each institution. Because data were collected retrospectively by study physicians in an anonymized manner, patient consent was not required.

## Results

### Patient characteristics

A total of 260 patients were included in the study, of whom 200 (77%) were pediatric patients (mean age: 5.7 years) and 60 (23%) were adult patients (mean age: 27.9 years). Table 1 presents patients’ demographics, schooling, and employment status at the time of varicella diagnosis. Half of the patients had mild disease (<50 skin lesions), 19 reported some varicella complication, and 10 (all of whom were children) were immunocompromised. Additional details on clinical characteristics are published elsewhere [26].

**Table 1:** Patient characteristics at varicella diagnosis.

Variable	Overall (N = 260)	Pediatric (n = 200)	Adults (n = 60)
**Males, N (%)**	150 (57.7%)	119 (59.5%)	31 (51.7%)
**Age, years, mean (SD)**	10.8 (10.8)	5.7 (4.4)	27.9 (7.4)
Employment status, N (%) *			
** Employed**	37 (14.2%)	0 (0.0%)	37 (61.7%)
** Unemployed**	169 (65.0%)	157 (78.5%)	12 (20%)
** Unknown**	54 (20.8%)	43 (21.5%)	11 (18.3%)
**Schooling status, N (%)**			
** Student full-time**	63 (24.2%)	52 (26.0%)	11 (18.3%)
** Student part-time**	5 (1.9%)	5 (2.5%)	0 (0.0%)
** Not in school**	101 (38.8%)	63 (31.5%)	38 (63.3%)
** Unknown**	91 (35.0%)	80 (40.0%)	11 (18.3%)

* Pediatric patients (<18 years) were assumed to have no employment.

As seen in Table 2, 99.6% (n = 259) of all patients reported a varicella-related outpatient visit, while 5.4% (n = 14) had an emergency department visit. Fourteen (5.4%) patients, 8 children and 6 adults, were hospitalized. Adults had a higher mean number of outpatient visits per patient compared with children (2.0 vs 1.3, *P*<0.001) but a shorter mean length of hospital stay (2.5 vs 5.4 days, *P* = 0.003). Thirty-one patients (11.9%) underwent at least 1 test or procedure, the most common (n = 17) being a complete blood count (54.8%). Almost all patients (95.8%) were prescribed at least 1 medication of any type. Overall, 20.8% of all patients were prescribed at least 1 antibiotic medication, and 46.5% were prescribed at least 1 antiviral medication (details published elsewhere) [26]. Adults were more likely to be prescribed at least 1 antiviral compared with children (96.7% vs 31.5%, *P*<0.001). Among the 48 pediatric patients who reported having missed school, the mean (SD) number of days lost was 5.8 (4.2) days, while the mean (SD) number of workdays lost reported by 32 adult patients was 7.4 (2.4) days (Table 2).

**Table 2:** Healthcare resource utilization and productivity loss.

Variable	Overall	Pediatric	Adult	*P*-value*
**Healthcare resource utilization**				
**Any outpatient visit, n (%)**	259 (99.6%)	199 (99.5%)	60 (100.0%)	<0.999
• Number of outpatient visits per patient, mean (SD) ^$^	1.4 (1.3)	1.3 (1.3)	2.0 (1.3)	<0.001
**Any ED visit, n (%)**	14 (5.4%)	14 (7.0%)	0 (0.0%)	0.081
**Any test/procedure**, n (%)	31 (11.9%)	19 (9.5%)	12 (20.0%)	0.028
• Number of tests/procedures per patient, mean (SD) ^$^	2.9 (3.4)	2.7 (2.5)	3.3 (4.5)	0.781
Any inpatient admission*	14 (5.4%)	8 (4.0%)	6 (10.0%)	0.071
• Length of hospital stay in days, mean (SD) ^$^	4.1 (3.3)	5.4 (3.9)	2.5 (0.8)	0.003
**Any medication use**	249 (95.8%)	190 (95.0%)	59 (98.3%)	0.466
• At least 1 antiviral, n (%)	121 (46.5%)	63 (31.5%)	58 (96.7%)	<0.001
• At least 1 antibiotic, n (%)	54 (20.8%)	38 (19.0%)	16 (26.7%)	0.199
**Any consultation with other specialists, n (%)**	7 (2.7%)	7 (3.5%)	0 (0.0%)	0.358
Productivity loss **				
**School days missed among pediatric patients, n**	48	48	NA	-
• Mean number of school days missed per child (SD) ^$^	5.8 (4.2)	5.8 (4.2)	NA	-
**Workdays lost among adult patients, n**	32	NA	32	-
• Mean number of workdays missed per adult (SD) ^$^	7.4 (2.4)	NA	7.4 (2.4)	-

ED, emergency department; HCRU, healthcare resource utilization; SD, standard deviation

* Comparison between pediatric and adult patients

$ Among those who reported HCRU or productivity loss

** We only considered school days missed among pediatric patients and workdays missed among adults when calculating the means. Thus, 2 children with reported missed workdays, 13 adults with reported missed school days, and 1 adult with 0 workdays were omitted from the analysis. For 5 pediatric hospitalized patients with unknown missed school days, missed school days were assumed to be equal to the length of the hospitalization.

#### Direct costs

The mean total direct medical cost for varicella across all patients (full sample) was approximately 33 USD/1,028 THB per patient (Table 3). The mean cost of outpatient visits (13 vs 8 USD/404 vs 258 THB, *P*<0.001) and all medications (9 vs 7/295 vs 207 THB, *P*<0.001) was significantly higher for adults compared with pediatric patients, while the mean cost of tests and procedures was higher for pediatric patients than for adult patients (6 vs 4 USD/176 vs 115 THB, *P* = 0.034).

**Table 3:** Mean cost per varicella patient, overall sample.

	Mean cost per patient across all patients (SD)						*P*-value*
Cost components	Overall (N = 260)		Pediatric (n = 200)		Adults (n = 60)		
	USD	THB	USD	THB	USD	THB	
**Direct costs**	33 (145)	1,028 (4,541)	33 (164)	1,027 (5,125)	33 (44)	1,031 (1,386)	<0.001 #
Outpatient visits	9 (13)	292 (398)	8 (12)	258 (390)	13 (13)	404 (408)	<0.001
ED visits	0 (2)	13 (56)	1 (2)	17 (63)	0 (0)	0 (0)	0.035
Hospitalizations	11 (77)	329 (2391)	12 (86)	363 (2696)	7 (24)	218 (759)	0.087
Tests or procedures	5 (44)	162 (1386)	6 (50)	176 (1566)	4 (12)	115 (390)	0.034
All medications	7 (26)	227 (807)	7 (29)	207 (914)	9 (6)	295 (190)	<0.001
** Indirect costs**							
Productivity loss	56 (102)	1,757 (3,193)	39 (91)	1,233 (2,853)	112 (117)	3,505 (3,641)	<0.001
** Total costs**	89 (207)	2,785 (6,466)	72 (221)	2,260 (6,908)	145 (138)	4,537 (4,304)	<0.001

ED, emergency department; SD, standard deviation. Note that the totals may not add up exactly due to rounding errors and foreign rate conversion

* Comparison between pediatric and adult patients

# P-value was calculated using non-parametric Wilcoxon rank sum test for skewed data and uses the medians for comparison. Although the mean direct costs appear similar in this case ($33 USD), the median direct cost (Q1 to Q3) was 271.9 (175.9 to 469.2) for pediatric patients (in THB) and 571.8 (407.2 to 918.6) for adults, resulting in a significant P-value.

Comparing mean direct medical costs specifically among pediatric and adult patients who utilized a healthcare resource (S2 Table), we found that pediatric patients had higher mean costs for varicella-related hospitalization (290 vs 70 USD/9,063 vs 2,175 THB, *P* = 0.017) but lower mean costs for outpatient visits (8 vs 13 USD/259 vs 404 THB, *P*<0.001) and varicella-associated medications (7 vs 10 USD/218 vs 300 THB, *P*<0.001) compared with adults. The mean cost of antibiotics among those prescribed at least 1 antibiotic to manage varicella was 9 USD/296 THB, while that for antivirals was 10 USD/300 THB among those prescribed at least 1 antiviral. The mean cost of antivirals among those prescribed at least 1 antiviral was higher among pediatric patients than adults (12 USD vs 7 USD/383 THB vs 210 THB, *P*<0.001). Additional details on medication use are provided elsewhere [26].

#### Indirect costs

Productivity losses (missed school or workdays as noted in the medical charts) were reported for one-quarter (24%) of pediatric patients and 50% of adult patients. The mean cost of productivity losses across all 260 patients was 56 USD/1,757 THB per varicella patient (Table 3). The mean indirect cost for all adult patients was 112 USD/3,505 THB, almost 3 times the mean caregiver cost for all pediatric patients, which was 39 USD/1,233 THB (*P*<0.001) (Table 3). When using 11 USD/331 THB, the minimum daily wage, for the sensitivity analysis, the total indirect cost decreased to 21 USD/656 THB, with mean indirect costs of 15 USD/469 THB and 42 USD/1,313 THB for pediatric and adult patients, respectively. Additionally, in the second sensitivity analysis, when the caregiver productivity loss for pediatric patients was presumed to be zero, the mean indirect cost per patient among all 260 patients decreased by 54%, from 56 USD/1757 THB to 26 USD/809 THB.

#### Total costs

The total mean cost (direct and indirect) per varicella patient was 89 USD /2,785 THB, with the total mean cost higher for adult than pediatric patients (145 vs 72 USD/4,537 vs 2,260 THB, *P*<0.001) (Table 3). The mean total cost (direct and indirect) was 61 USD/1904 THB per outpatient case and 584 USD/18,265 THB per inpatient case. Indirect costs accounted for 63% of the total cost per patient (54% for pediatric patients, 77% for adult patients).

Additionally, for our exploratory analysis, we estimated that the annual national burden of varicella in Thailand could be approximately 33.4 million USD or 1,042.6 million THB (assuming a 1.4% hospitalization rate for varicella), and approximately 45.1 million USD or 1,409.3 million THB (assuming a 6% hospitalization rate for varicella) [17,18] (S1 Fig).

## Discussion

This study provides a comprehensive assessment of HCRU and costs associated with varicella among children and adults seeking healthcare in Thailand and demonstrates the substantial economic burden of varicella. The total mean per patient cost for managing varicella in a healthcare setting was 89 USD/2,785 THB.

The mean number of varicella-related outpatient visits reported among pediatric patients (1.3 visits) is in line with estimates from the other middle-income countries (1.1 to 2.2 visits per case) and from the literature review of varicella patients of all ages (0.04 to 2.2 visits per case) [9,19]. The mean length of hospital stay of 5.4 days reported for the pediatric population in our study is also consistent with that in the MARVEL study (3.6 to 6.8 days) [9] but slightly higher than that reported among children in Hong Kong (3.7 days) [3,32]. The only estimate from Thailand is from a previous study by Vandepitte et al., which reported the median length of stay to be 6 days (interquartile range, 3–9 days) among 101 Thai children hospitalized for varicella [20]. A systematic literature review of varicella in the Asia-Pacific region also found the varicella-related average length of hospital stay in the general population (all ages) to range from 3.3 to 7.4 days, which aligns with our results (mean 4.1 days, all ages) [3]. A recent systematic literature review reported the average length of stay among patients with uncomplicated varicella specifically to range from 2.1 and 4.7 days [19]. Outpatient visits and hospitalizations not only highlight the direct burden of varicella but also contribute to indirect costs due to potential work loss.

Among children 0–17 years (n = 48) who reported any missed school days in our study, the mean number of days lost was 5.8 days per child. In comparison, a Spanish study of children aged 0–14 years with varicella before the introduction of UVV found the mean school days lost to be 6.2 days [33]. Another pre-UVV study from the United States (US) reported a mean of 7.6 days lost among children 5–17 years [34]. Differences among missed school days reported by studies may reflect the varying age range of children or differences in return-to-school policies following varicella infection. Missed school days may impose a greater burden on adolescents and young adults and also affect quality of life, impacts that are not captured in this study [22,35].

Productivity losses account for 63% of total per-patient varicella costs, including 77% of total per-patient costs in adults, compared with 54% in children. The lower proportion of indirect costs among children could possibly be due to the availability of alternative childcare options (e.g., grandparents) in Thailand, minimizing time off by parents, as well as reporting issues. We assumed that parents had to take a day off for every school day missed and that those who did not report any missed school days/workdays did not have any productivity loss. But this approach may either overestimate the true productivity loss (e.g., the working parent did not have to take time off because a stay-at-home parent or grandparent provided childcare for the child missing school) or underestimate it (due to incomplete reporting of productivity loss in the medical charts). Hence, we conducted a conservative sensitivity analysis (where parents were assumed to have zero productivity loss), which found indirect costs accounted for 44% of the total cost per patient.

Adult patients missing work (n = 32) in our study reported a mean productivity loss of 7.4 workdays per patient. Productivity loss averaged across all adults was $112 per patient, contributing to substantial indirect costs among adults. This may have significant economic implications in Thailand [23,24] and highlights the need to include indirect costs to capture the full burden of disease in economic analyses [22]. Studies in Australia and the US found a greater number of missed workdays reported by mothers than fathers, due to mothers being more likely to take time off than fathers [34,36]. Our study did not account for such gender differences in the analysis.

Our exploratory analysis estimated the total cost of varicella management nationally could range from 33.4 to 45.1 million USD (or 1,042.6 million to 1,409.3 million THB) annually in Thailand, depending upon the hospitalization rate used [17,18]. However, data were primarily collected from Bangkok; hence, the generalizability of these results to the entire Thai population could be limited. Future research is needed to collect data from a geographically representative sample to better estimate the national burden of disease.

Our study is the first to assess the burden of varicella among adults in Thailand. Looking at direct costs only, Vandepitte et al. found the median (IQR) costs of hospitalization of Thai children with varicella to be 330 USD (139–1013 USD) [20]. Our estimates of hospitalization costs were also lower compared with costs in high-income countries in the Asia-Pacific region, such as Australia (3414 USD), Hong Kong (3157 USD), and New Zealand (1499 USD) in 2017 USD [3,10,12,15]. The mean total cost (direct and indirect) was 61 USD per outpatient case and 584 USD per inpatient case in our study. Our total costs are lower than the mean total cost (direct and indirect) per child from the multi-country MARVEL study (for outpatients: ranging from 98 USD in Peru to 323 USD in Argentina; for inpatients: ranging from 736 USD in Hungary to 5786 USD in Mexico; 2015 USD) [9,37]. However, this could reflect differences in healthcare systems and cost structures among the countries, differences in annual wages, and differences in approaches to calculating productivity costs. With the universal health coverage system in Thailand, most of the direct medical costs related to varicella are borne by the national government [38,39].

The implementation of UVV has led to significant reductions in varicella cases, hospitalizations, and costs in the Asia-Pacific region and globally [1,4–7,40–42]. For example, the introduction of UVV led to a 2.9- to 3.8-fold reduction in varicella disease burden in Taiwan [3,43]. Similarly, the implementation of a UVV program in the US over 25 years led to a 97% decrease in varicella disease, with net societal savings of $23.4 billion [7]. UVV could be considered a possible strategy to reduce the clinical and economic burden of varicella in Thailand.

One of the strengths of our study is that we selected patients based on month and year of index date to account for any seasonal variation in costs. We also drew our sample from both private and public hospitals and included both pediatric and adult patients in our sample to reflect the disease seroprevalence in Thailand [23,24]. Additionally, the ~5% (14/260) hospitalization rate of inpatients recruited in our convenience sample aligns with the 1.4%-6% hospitalization rates from previous studies [17,18]. Results from this study provide updated economic perspectives that could inform the development of public health interventions and immunization policies related to varicella and varicella vaccination.

Limitations include possible inaccurate or incomplete information in patient records affecting data quality. All 4 sites were in the Bangkok region, and the Bangkok metropolitan region is home to almost a quarter (22.2%) of the national population [25]. However, the generalizability of these results to the rural population or to the entirety of Thailand could be limited. By including patients from different age groups (pediatric and adult) and settings (public and private) in our study sample, we tried to increase the generalizability of our study. Our study included only patients who sought healthcare and may not reflect costs among all varicella patients, including those with milder symptoms who did not seek medical consultation. Additionally, the small number of inpatients may underestimate the mean cost across all patients. Similarly, the disease severity and immunocompromised status of patients in our sample may have influenced costs. Only 24% of children and 53% of adults had missing school days and workdays noted in their charts, and it is likely that this information was not captured for all patients in the medical charts, impacting our results. We also assumed that school days lost will be equal to productivity loss among caregivers, which may not always be true. We tried to mitigate this bias by performing sensitivity analysis with the most conservative estimates. However, our sample size was still robust, with both pediatric and adult patients included. Assumptions were made regarding wages and productivity loss that may impact the results. Additional non-medical direct costs (i.e., out-of-pocket expenses, travel) incurred by patients and staff costs were not accounted for in our analysis, leading to possible underestimation of the costs. Finally, our study data collection was subject to feasibility, budget, and time constraints related to the COVID-19 pandemic; data collection for this study was stopped early, impacting the final sample size, as healthcare providers redirected resources to the pandemic.

## Conclusion

There is substantial economic burden associated with varicella among patients seeking healthcare in Bangkok, Thailand. Our study estimated higher total costs for adult than pediatric patients. Indirect costs associated with productivity loss were the primary driver of total costs for varicella management, especially for adults. This study may be of interest to policymakers in Thailand and neighboring countries considering UVV as a tool to reduce the economic burden associated with varicella.

## Supporting information

S1 Fig Estimated national annual economic burden of varicella in Thailand (2020 USD). (DOCX)

S1 Table Source for unit costs for healthcare resource utilization, tests, procedures, and medications. (DOCX)

S2 Table Healthcare resource cost among those with resource use (by type). (DOCX)
