# Supplementary material for: Economic burden of varicella in Bangkok, Thailand: A multicenter medical chart review study
Source: PLOS Glob Public Health. 2024 Jun 12;4(6):e0003099. doi: 10.1371/journal.pgph.0003099 (PMC11168696; doi:10.1371/journal.pgph.0003099)
Supplement: S1 Fig — (DOCX) [file pgph.0003099.s001.docx]

**S1 Fig**

**Additional information on the calculation of the national burden of varicella**

**Estimated national annual economic burden of varicella in Thailand (2020 USD)**


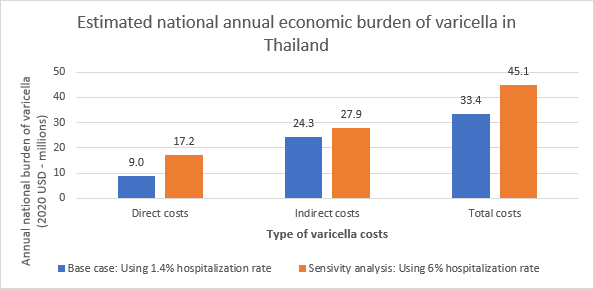


Annual costs for varicella in Thailand were calculated by estimating the annual number of inpatient and outpatient cases in Thailand, multiplying each by the mean cost per inpatient or outpatient case calculated from our study, and then taking the sum.

Due to a lack of availability of good incidence data in Thailand, we used the pre-UVV data varicella incidence rate of 699.7 per 100,000 population from Tseng et al., which used the Taiwan National Health Insurance database, covering more than 96% of the population and hence is a good reflection of patients seeking medical help for varicella in Taiwan [1]. This was applied to the total reported population of Thailand in 2020 (n=69,799,978) [2] to estimate the annual number of total varicella cases in Thailand seeking medical care for varicella. We used the varicella-related hospitalization rates of 1.41% and 6% from Taiwan [1, 3] to estimate the annual number of inpatient varicella cases in Thailand for base case and sensitivity analyses, respectively. The number of outpatient cases was then calculated by subtracting the number of inpatient varicella cases from the total number of varicella cases.

**References**

1. Tseng HF, Tan HF, Chang CK. Varicella epidemiology and cost-effectiveness analysis of universal varicella vaccination program in Taiwan. Southeast Asian J Trop Med Public Health. 2005;36(6):1450-8. Epub 2006/04/14. PubMed PMID: 16610647.

2. The World Bank. Population, total - Thailand: World Bank Group; 2022 [cited 2022 July 19]. Available from: <https://data.worldbank.org/indicator/SP.POP.TOTL?locations=TH&view=chart>.

3. Lin YH, Huang LM, Chang IS, Tsai FY, Chang LY. Disease burden and epidemiological characteristics of varicella in Taiwan from 2000 to 2005. J Microbiol Immunol Infect. 2009;42(1):5-12. Epub 2009/05/09. PubMed PMID: 19424552.
