# Supplementary material for: Economic burden of varicella in Bangkok, Thailand: A multicenter medical chart review study
Source: PLOS Glob Public Health. 2024 Jun 12;4(6):e0003099. doi: 10.1371/journal.pgph.0003099 (PMC11168696; doi:10.1371/journal.pgph.0003099)
Supplement: S1 Table — (DOCX) [file pgph.0003099.s002.docx]

**S1 Table**

**S1 Table. Source for unit costs for healthcare resource utilization, tests, procedures, and medications**

| **Healthcare resource** | **Unit cost source** |
| --- | --- |
| - Inpatient hospitalization (per day) | Reported by physicians in our survey |
| - ED visit | Reported by physicians in our survey |
| - Outpatient office/clinic visit | Reported by physicians in our survey |
| **Tests and procedures** |  |
| - Blood culture; CT scan; CT brain; CT chest; CSF culture; ECG; MRI; Sputum culture; Urine culture; VZV culture; VZV DNA test; VZV Antibody test; X-ray | Reported by physicians in our survey |
| - Stool culture; Skin culture; Lesion fluid analysis; Blood urea nitrogen test (BUN); CBC; Creatinine; Dengue NS1 antigen test; EBV viral load; ESR (erythrocyte sedimentation rate); Electrolytes; Influenza test; Liver function test; Malaria; SGOT (liver function test); SGPT (liver function test); Type-specific HSV serologic test; Tzanck smear; Urine analysis | Other sources |
| **Medication** |  |
| - Acyclovir (200 mg tablet) - per pill; Acyclovir (400 mg tablet); Acyclovir (800 mg tablet); Acyclovir (IV) - per mg; Acyclovir (oral); Topical mupirocin; Chloramphenicol ointment; Prednisolone cream; Triamcinolone acetonide; Acetaminophen (325 mg tablet); Acetaminophen (oral) - per bottle; Ibuprofen (200 mg); Ibuprofen (400 mg); Ciprofloxacin (250 mg tablet) ; Ciprofloxacin (500 mg tablet) Azithromycin (250 mg tablet); Cephalexin (250 mg tablet); Cephalexin (500 mg tablet); Dicloxacillin (250 mg tablet); Dicloxacillin (500 mg tablet); Domperidone (10 mg tablet); Cefditoren (400 mg tablet) - per mg; Clindamycin (150 mg tablet); Clindamycin (300 mg tablet); Dextromethorphan (15 mg tablet); Amikacin (IV)- per mg ; Ceftazidime (IV); Ceftriaxone (IV); Dicloxacillin (oral); Augmentin/Amoxicillin-clavulanate (1000 mg tablet); Augmentin/Amoxicillin-clavulanate (250 mg tablet); Amoxicillin-clavulanate; Xylocaine (oral); Oral antihistamine (10 mg tablet); Oral antihistamine (60 mL); Bactrim | DMSIC |
| - Acyclovir (topical) - per tube; Herpes lotion; Topical anti-itch cream; Piperacillin/Tazobactam (IV) - per bottle; Bromhexine (8 mg tablet); Bromhexine (mL) - per bottle; Multivitamin drop (15 mL bottle); Normal saline (300 mL topical solution); Normal saline (15 mL nasal spray); Valacyclovir (500 mg tablet); Betadine/Povidone-iodine; Fucidin cream; Oral rehydration solution; Topical moisturizing cream - per bottle; Amitriptyline (10 mg tablet); Carbocisteine - per bottle - 250 mg/5mL; Carbocisteine (tablet) - per mg; Ferrous sulfate - per bottle; IV fluid; Fluoroquinolone (200 mg Norxacin tablet; 500 mg Ciproxyl tablet); Fluimucil (acetylcysteine) (200 mg); Hibiscrub | Physician-reported |

CBC, complete blood count; CSF, cerebrospinal fluid; CT, computed tomography; DMSIC, Drug and Medical Supply Information Center; EBV, Epstein-Barr virus; ECG, electrocardiogram; ED, emergency department; HCRU, healthcare resource utilization; HSV, herpes simplex virus; IV, intravenous; MRI, magnetic resonance imaging; SGOT, serum glutamic-oxaloacetic transaminase; SGPT, serum glutamic-pyruvic transaminase; THB, Thai baht; VZV, varicella zoster virus.
