# Supplementary material for: Economic burden of varicella in Bangkok, Thailand: A multicenter medical chart review study
Source: PLOS Glob Public Health. 2024 Jun 12;4(6):e0003099. doi: 10.1371/journal.pgph.0003099 (PMC11168696; doi:10.1371/journal.pgph.0003099)
Supplement: S2 Table — (DOCX) [file pgph.0003099.s003.docx]

**S2 Table**

**S2 Table. Healthcare resource cost among those with resource use (by type)**

|  |  | **Average cost per resource user** | | ***P*-value** |
| --- | --- | --- | --- | --- |
| Resource type | # of users | Mean (SD) | Mean (SD) |  |
| **Outpatient visits** | **n** | **USD** | **THB** |  |
| Overall | 259 | 9 (13) | 293 (398) | - |
| Pediatric | 199 | 8 (12) | 259 (390) | <0.001 |
| Adult | 60 | 13 (13) | 404 (408) |  |
| **ED visits** | **n** | **USD** | **THB** |  |
| Overall | 14 | 8 (0) | 247 (0) | - |
| Pediatric | 14 | 8 (0) | 247 (0) | - |
| Adult | 0 | 0 (0) | 0 (0) | - |
| **Hospitalizations** | **n** | **USD** | **THB** |  |
| Overall | 14 | 196 (279) | 6,111 (8,709 ) | - |
| Pediatric | 8 | 290 (345) | 9,063 (10,790) | 0.017 |
| Adult | 6 | 70 (41) | 2,175 (1,297) |  |
| **Tests/Procedures** | **n** | **USD** | **THB** |  |
| Overall | 31 | 43 (124) | 1355 (3861) | - |
| Pediatric | 19 | 59 (156) | 1847 (4885) | 0.414 |
| Adult | 12 | 18 (23) | 577 (726) |  |
| **All medications** | **n** | **USD** | **THB** |  |
| Overall | 249 | 8 (26) | 237 (823) | - |
| Pediatric | 190 | 7 (30) | 218 (937) | <0.001 |
| Adult | 59 | 10 (6) | 300 (187) |  |
| **All antibiotics** | **n** | **USD** | **THB** |  |
| Overall | 54 | 9 (8) | 296 (250) | - |
| Pediatric | 38 | 9 (8) | 294 (266) | 0.677 |
| Adults | 16 | 10 (7) | 300 (218) |  |
| **All antivirals** | **n** | **USD** | **THB** |  |
| Overall | 121 | 10 (34) | 300 (1073) | - |
| Pediatric | 63 | 12 (48) | 383 (1486) | <0.001 |
| Adults | 58 | 7 (2) | 210 (58) |  |

ED, emergency department; SD, standard deviation.
